# Prokaryotic Community Distribution along an Ecological Gradient of Salinity in Surface and Subsurface Saline Soils

**DOI:** 10.1038/s41598-017-13608-5

**Published:** 2017-10-17

**Authors:** Kehui Xie, Yong Deng, Shaocun Zhang, Wenhao Zhang, Jianrong Liu, Yulong Xie, Xuze Zhang, He Huang

**Affiliations:** 1 School of Chemical Engineering and Technology, Tianjin University, Tianjin, 300350 People’s Republic of China; 2 School of Chemistry and Chemical Engineering, Qinghai University for Nationalities, Xining, 810007 People’s Republic of China

## Abstract

Salinity effects on microbial communities in saline soils is still unclear, and little is known about subsurface soil microbial communities especially in saline or hypersaline ecosystems. Here we presented the survey of the prokaryotic community in saline soils along a salinity gradient (17.3–148.3 dS/m) in surface (0–10 cm) and subsurface (15–30 cm) saline soils of Qarhan Salt Lake, China. Moreover, we compared them with three paired nonsaline normal soils. Using the high-throughput sequencing technology and several statistical methods, we observed no significant community difference between surface soils and subsurface soils. For environmental factors, we found that TOC was the primary driver of the prokaryotic community distribution in surface saline soils, so was pH in subsurface saline soils. Salinity had more effects on the prokaryotic community in subsurface saline soils than in surface saline soils and played a less important role in saline soils than in saline waters or saline sediments. Our research provided references for the prokaryotic community distribution along a salinity gradient in both surface and subsurface saline soils of arid playa areas.

## Introduction

Recently, people began to realize the importance of halophilic microorganisms in the industrial biotechnology because of their advantages, such as less fresh water consumption, low energy, continuous production, and low fixed capital investment^1^. And many halophilic hydrolases secreted by halophiles, such as amylases, lipases, and cellulases, are promising for industrial applications under diverse conditions due to their polyextremophilicity^2,3^. Hypersaline ecosystems, which represent a wide variety of ecosystem types, such as salt lakes, salt flats, playas, solar salterns, and ancient salt deposits, are globally distributed^4–6^. Halophilic and halotolerant microorganisms thrive broadly in these ecosystems; therefore, it is essential and valuable to make in-depth investigations on the microbial communities of these ecosystems.

A previous comprehensive analysis based on sequencing data from many researches of diverse physical environments had proved that salinity, rather than extremes of temperature, pH, or other environmental factors, mainly determined microbial communities^7^. However, the comprehensive analysis contained only saline waters or sediments in aquatic environments and no saline soils were included. Moreover, while the salinity has been characterized as one of primary factors of the microbial community distribution in aquatic environments, the extent of salinity effects on microbial communities in saline soils were still unclear^8^. And, few studies have focused on the correlation between salinity and the microbial community distribution in saline soils^8–10^. Therefore, more studies need to be done to clarify the extent of salinity effects on microbial communities in saline soils.

Present studies of microbial communities in soils focused exclusively on surface (0–10 cm) soils, and several studies^11–15^ have focused on microbial communities in both surface and subsurface nonsaline soil layers. Though microorganisms in surface soils were more dense and active collectively^12^, those in subsurface saline soils can also play an important role on nutrition cycling, ecosystem function, and soil respiration^16,17^. And, for microbial communities in saline or hypersaline soils, most previous studies^9,10,18,19^ just focused on the microbial community in surface soil layers (0–10 cm). No previous reports of microbial communities in subsurface saline soil layers (15–30 cm) seems to exist.

The Qaidam Basin is located in the northeast of Qinghai–Tibetan Plateau, China. There are twenty seven salt lakes in the basin; the playas and salt lakes take up about a quarter of the whole basin area^20^. Among all 27 lakes, Qarhan Salt Lake is the largest playa, where 70% of the total thickness of the upper salt-bearing deposits is composed of the halite layers^20^. It is the largest industrial base for potassium fertilizer in China, and it is also rich in Mg, Li, and many other salt resources. Its unique significances in scientific research, resource exploitation, and environmental protection are increasingly being recognized^21^. Many previous studies have focused on the prokaryotic communities in waters^22–25^ or sediments^26,27^ of the lakes in Qinghai-Tibetan Plateau, and they all observed that salinity was the primary driver of the microbial community distribution. However, the prokaryotic community distribution in saline soils and its correlations with environmental factors, especially the salinity, are still uncharacterized in this area.

The purposes of this research are: 1) to clarify the prokaryotic community distribution along several environmental gradients, especially the salinity gradient, in both surface and subsurface saline soils; 2) to characterize the prokaryotic community composition in saline soils and compare with that in nonsaline normal soils; 3) to ascertain the microbial resources for further exploitations.

## Results

### Physical and chemical properties of soils

The 23 saline soils from Qarhan Salt Lake showed a wide salinity gradient from 17.3 dS/m to 148.3 dS/m, a pH gradient from 7.36 to 8.92, a TOC (total organic carbon) gradient from 4.19% to 21.48%, and a water content gradient from 1.1% to 32.3% (Table 1).The data indicated a wide environmental range, especially the salinity range. In eight paired surface-subsurface saline soils, surface soils had higher salinity (except SS1 and SD1, SS8 and SD8), lower water contents (except SS4 and SD4), and lower pH values (except SS7 and SD7, SS8 and SD8) than subsurface soils (Table 1). The six nonsaline normal soils encompassed few variations of environmental factors compared with saline soils. Spearman’s rank correlation was conducted between EC (electrical conductivity) and other environmental factors (Supplementary Table S2). EC had significant (*P* < 0.05) positive correlations with concentrations of Na, Ca, Mg and significant negative correlations with other factors.

**Table 1:** Geographical, physical, and chemical properties of soils. ND: not detected.

Sample ID	pH	EC (dS/m)	TOC (%)	WC (%)	K (g/kg)	Ca (g/kg)	Mg (g/kg)	Na (g/kg)	locations (N,E)	Altitude (m)
SS1	7.81	119.4	5.44	12.00	3.99	11.02	1.55	18.75	36°49′32″, 95°10′15″	2675
SD1	8.09	148.3	4.19	13.90	4.49	10.00	1.48	18.41		
SS2	7.67	74.5	5.36	6.60	4.69	5.75	1.44	18.19	36°49′35″, 95°10′07″	2674
SD2	7.85	67.5	5.52	11.10	4.01	11.00	1.41	16.24		
SS3	7.78	123.1	4.97	6.00	3.85	10.52	1.46	18.55	36°49′34″, 95°10′06″	2673
SD3	7.93	46.9	6.14	15.00	4.01	11.01	1.43	14.11		
SS4	7.8	83.7	6.09	8.90	4.45	7.99	1.65	18.36	36°49′32″, 95°10′06″	2677
SD4	7.87	58.6	5.13	7.10	3.89	10.77	1.43	15.44		
SS5	7.8	138.4	4.20	5.80	3.74	10.02	1.43	18.80	36°49′31″, 95°10′07″	2674
SD5	7.83	84.1	5.29	8.40	3.95	11.02	1.38	17.37		
SS6	7.88	92.6	5.13	13.20	4.13	9.19	1.48	17.91	36°49′29″, 95°10′07″	2678
SD6	8.02	34.4	6.22	14.70	3.99	11.02	1.41	14.04		
SS7	8.74	109.3	11.67	1.10	3.82	33.24	2.06	18.13	36°32′30″, 94°58′51″	2744
SD7	8.64	103.6	8.71	15.50	3.65	35.94	2.07	17.91		
SS8	8.82	75.5	21.18	1.60	4.08	36.89	2.08	17.70	36°35′40″, 94°59′57″	2742
SD8	8.74	122.2	21.48	5.20	4.17	6.28	1.50	18.72		
US1	7.36	61.4	4.74	ND	4.15	8.97	1.40	15.59	36°49′32″, 95°10′12″	2676
US2	7.8	52.1	5.06	5.80	4.15	8.89	1.41	17.42	36°49′34″, 95°10′11″	2676
US3	7.98	81.7	5.21	ND	4.51	5.84	1.42	19.02	36°49′39″, 95°10′25″	2693
UD1	8.13	47.6	6.14	32.30	3.96	10.79	1.37	15.38	36°49′27″, 95°10′08″	2678
UD2	7.97	64.7	5.36	ND	3.90	10.91	1.35	17.42	36°49′23″, 95°10′20″	2667
UD3	8.63	17.3	7.00	15.90	4.06	9.07	1.38	13.70	36°34′06″, 94°59′57″	2731
UD4	8.92	42.6	5.05	1.10	4.11	8.77	1.37	15.96	36°36′43″, 95°00′54″	2703
NS1	8.8	1.2	15.64	11.40	4.59	8.01	1.39	15.68	39°00′12″, 117°16′41″	2.7
ND1	8.9	1.1	12.99	20.30	4.25	4.69	1.22	10.35		
NS2	8.5	0.2	16.50	17.60	4.61	6.03	1.40	16.55	39°04′48″, 117°09′53″	5
ND2	8.7	0.3	20.94	16.10	4.62	3.88	1.15	15.25		
NS3	9.3	0.2	22.50	12.70	4.53	1.60	1.15	11.34	39°04′48″, 117°09′53″	3.2
ND3	9.1	0.5	17.02	17.40	4.61	3.38	1.24	11.47		

Abbreviations: “SS”, “SD”, “US”, “UD”, “NS”, and “ND” represent shallow saline soil (surface saline soil), deep saline soil (subsurface saline soil), unpaired surface saline soil, unpaired subsurface saline soil, surface normal soil and subsurface normal soil, respectively. EC: electrical conductivity, TOC: total organic carbon, WC: water content.

### Statistics for 16S rDNA sequencing data

After quality filtering and chimera checking, 1,693,891 effective tags were obtained with an average length of 254 bp from all 29 soil samples, of which 1,335,650 tags belonged to 23 saline soil samples from Qarhan Salt Lake and 358,241 tags to 6 normal soil samples from Tianjin, China (Supplementary Table S1). We also summarized Q20, Q30, GC%, and the number of effective tags (Supplementary Table S1). The number of effective tags of each sample ranged from 47,504 to 67,321 among saline soils and from 51,102 to 64,426 among normal soils. The effective tags of each sample were subject to further analysis.

**Figure 1:**
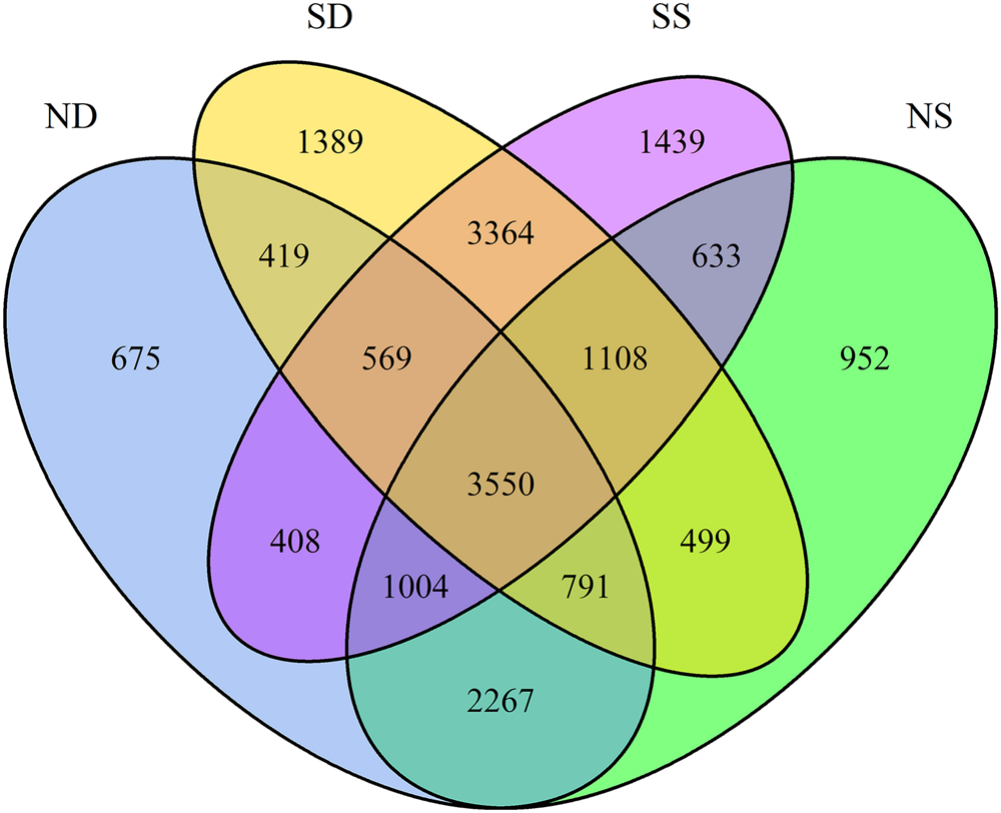
Venn diagrams of OTUs included in four groups of soils. We grouped all soil samples into NS, ND, SS, SD, representing shallow normal soil (surface normal soil), deep normal soil (subsurface normal soil), shallow saline soil (surface saline soil), deep saline soil (subsurface saline soil), respectively.

For OTU picking, we obtained 56,541 OTUs with a mean of 2,458 ± 904 (s.d.) OTUs from 23 saline soil samples and 33,877 OTUs with a mean of 5,646 ± 607 OTUs from six normal soil samples (Supplementary Table S1). The mean of OTUs in normal soils was more than twice as many as that in saline soils, which indicated low microbial diversity in saline soils. The sum of OTUs in saline soil samples (surface saline soils: 12075; subsurface saline soils: 11689) was higher than that in normal soils (surface normal soils: 10804; subsurface normal soils: 9683), and the sum of OTUs in surface soils was higher than in subsurface soils of both saline soils and normal soils (Fig. 1). Moreover, shared OTUs took up more than 70% between surface normal soils and subsurface normal soils (7612/10804), surface saline soils and subsurface saline soils (8591/12075). And shared OTUs took up about 50% between surface normal soils and surface saline soils (6295/12075), subsurface normal soils and subsurface saline soils (5329/11689) (Fig. 1). The number of OTUs shared by all four groups was 3550.

### Prokaryotic community composition and alpha diversity analysis

**Figure 2:**
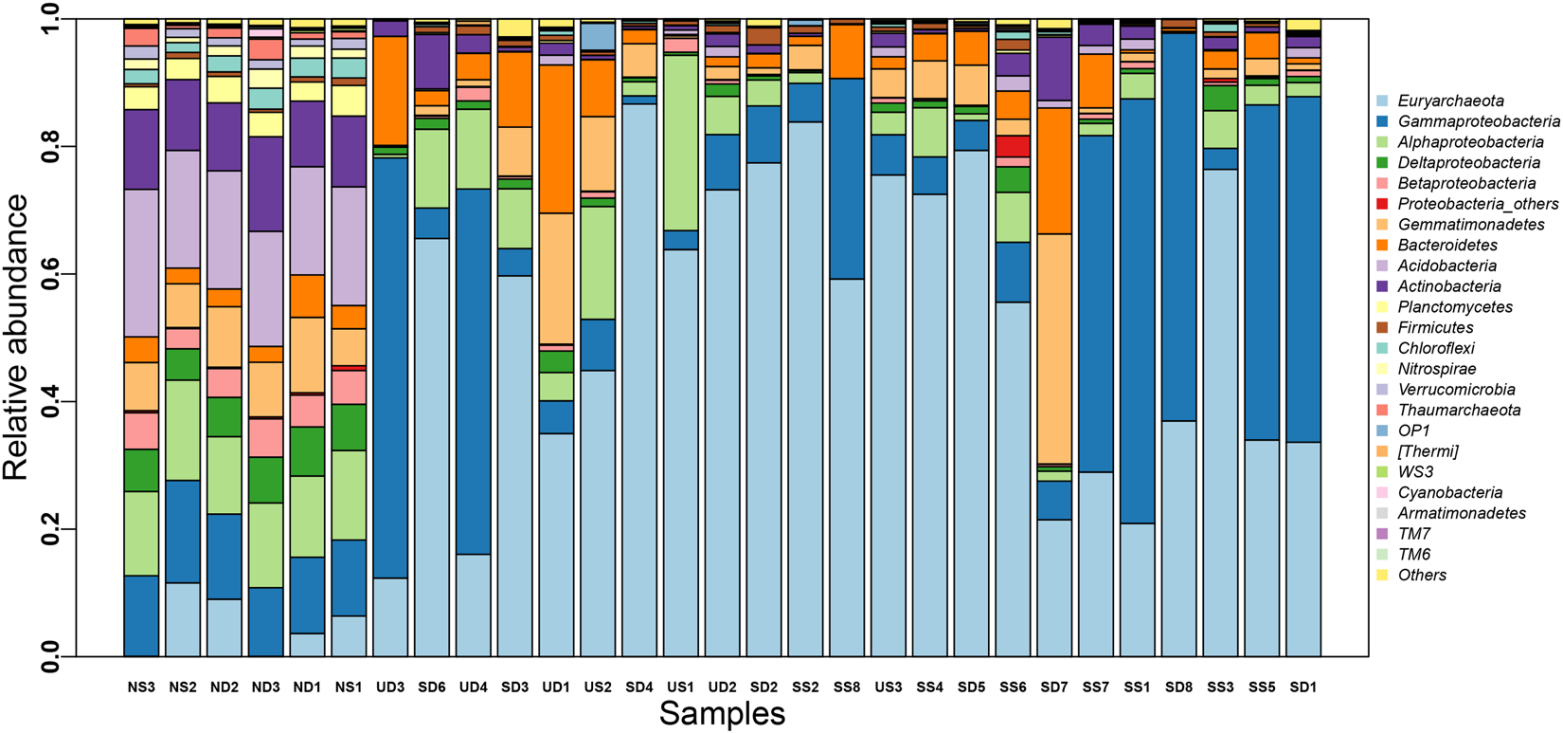
Relative abundance of 19 most predominant phyla (including four classes in *Proteobacteria*) in all samples sorted from left to right with increasing salinity.

*Archaea* and *Bacteria* accounted for an average percentage of 52.8% (12.3–86.7%) and 47.2% (13.3–87.7%) of all 1,335,650 effective tags respectively in saline soils, with a huge difference compared with 6.8% (2.9–12.1%), 93.2% (87.9–97.2%) in normal soils. Detailed relative abundance of each phylum/class were summarized in Supplementary Table S3. Saline soils showed low microbial complexity compared with normal soils, and there were high percentages of *Euryarchaeota* in most saline soil samples (Fig. 2).

For *Bacteria* at phylum level, *Proteobacteria* (35.1% ± 23.8% in surface saline soils, 23.5% ± 22.9% in subsurface saline soils), *Bacteroidetes* (4.3% ± 2.9%, 5.6% ± 3.9%), and *Gemmatimonadetes* (2.3% ± 1.8%, 7.3% ± 3.1%) were the three most abundant phyla in saline soils (Supplementary Fig. S1). And within *Proteobacteria*, *Gammaproteobacteria* (28.5% ± 25.7% in surface saline soils, 18.3% ± 2.5% in subsurface saline soils) were the most abundant class, which accounted for more than half (68.7% ± 31.0%, 59.4% ± 29.2%) of *Proteobacteria*, followed by *Alphaproteobacteria* (4.0% ± 2.9%, 4.1% ± 3.1%), *Deltaproteobacteria* (1.5% ± 1.3%, 0.9% ± 0.5%), and *Betaproteobacteria* (0.6% ± 0.5%, 0.3% ± 0.3%). In normal soils, *Proteobacteria* (39.3% ± 0.8% in surface normal soils, 37.2% ± 0.7% in subsurface normal soils), *Acidobacteria* (17.8% ± 0.8%, 20.1% ± 2.7%), *Actinobacteria* (11.9% ± 2.5%, 11.6% ± 0.8%) were the three most abundant phyla. And within *Proteobacteria* in normal soils, *Alphaproteobacteria* (14.3% ± 1.3%, 12.7% ± 0.6%) were the most abundant phylum, followed by *Gammaproteobacteria* (13.5% ± 2.2%, 12.0% ± 1.3%), *Deltaproteobacteria* (6.3% ± 1.2%, 7.0% ± 0.8%), and *Betaproteobacteria* (4.7% ± 1.4%, 5.2% ± 0.8%) (Supplementary Fig. S1). Standard deviations of phyla/classes in saline soils were much higher than in normal soils, indicating wide variations of phyla/classes in saline soils.

For *Archaea*, almost all effective tags were assigned to *Halobacteria* of *Euryarchaeota* in saline soils. But in normal soils, about 25% of *Archaea* tags were assigned to *Thaumarchaeota* except *Halobacteria* of *Euryarchaeota* (Fig. 2).

ANOSIM results showed significant prokaryotic community differences between surface saline soils and surface normal soil (*R* = 0.37, *P* < 0.05), subsurface saline soils and subsurface normal soils (*R* = 0.36, *P* < 0.05). To further identify the phyla/classes which contributed primarily to the community variations, we conducted SIMPER analysis between surface normal soils and surface saline soils, subsurface normal soils and subsurface saline soils (Supplementary Table S4). The overall community dissimilarity (the sum of contribution) between surface normal soils and surface saline soils was 68.9%, and the value was 72.1% between subsurface normal soils and subsurface saline soils at the phylum/class level. *Euryarchaeota* made the greatest contribution to the community variations in both surface and subsurface soils between saline soils and normal soils. *Gammaproteobacteria* and *Deltaproteobacteria* played a more important role in differentiating the surface soil community, but *Alphaproteobacteria* contributed more in subsurface soil community (Supplementary Table S4).

**Figure 3:**
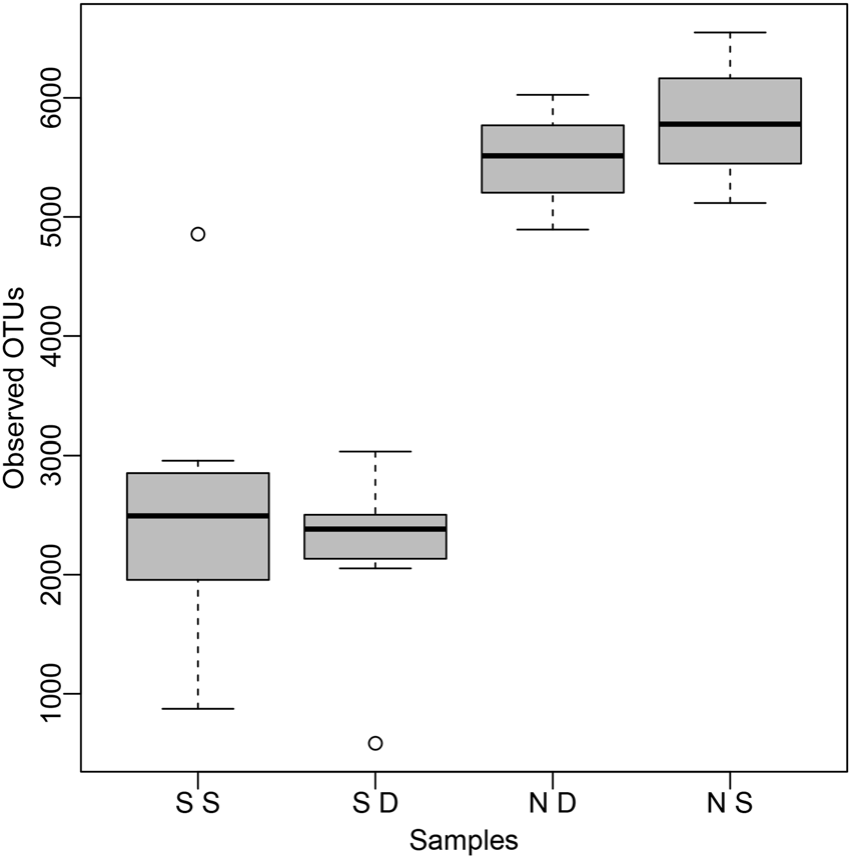
Alpha diversity boxplot among four groups same as Fig. 1 but without seven unpaired samples to remove the influence of environmental factors. The values of Observed OTUs were achieved after normalization (47504 tags per sample). The three lines of the box from bottom to top represents the first quartile (Q1), the second quartile (the median), and the third quartile (Q3), respectively; the two ends of the whiskers represent the minimum and maximum of the data within a group, respectively. Outliers were the values beyond the range (Q1 − 2 IQR, Q3 + 2 IQR). IQR (interquartile range) = Q3 − Q1.

As it is shown in Fig. 3, microorganisms in surface soils are richer than those in subsurface soils for both saline soils and normal soils. Surface soils had more OTUs than their corresponding subsurface soils except SS1 and SD1, SS2 and SD2 (Supplementary Table S1). Though we observed more *Gammaproteobacteria* in surface saline soils than in subsurface saline soils, and *Bacteroidetes* and *Gemmatimmonadetes* in surface saline soils were less than those in subsurface saline soils by summarizing prokaryotic community compositions in all paired saline soils (Supplementary Fig. S1). ANOSIM results showed no significant (*P* = 0.8 for normal soils and *P* = 0.26 for paired saline soils) prokaryotic community difference between surface soils and subsurface soils, and Student’s t-test results of each phylum/class were also unsignificant (*P* > 0.05) between the two groups in both saline soils and normal soils.

Rarefaction curves of most samples (except UD3, SS8, and SD8) did not reach an obvious asymptote, indicating that there were still many undetermined species (Supplementary Fig. S2). Chao1 estimators suggested that our sequencing efforts contained about 54.8% ± 7.8% of the estimated diversity for saline soils and about 64.8% ± 1.3% for normal soils (Supplementary Table S1), which indicated larger proportions of unknown species in saline soils than in normal soils. The curves of six normal soils were obviously above curves of 23 saline soils, demonstrating low microbial diversities in saline soils (Supplementary Fig. S2). Shannon diversity index (5.9 ± 1.5 in saline soils and 10.2 ± 0.3 in normal soils) also verified that (Supplementary Table S1).

### Correlations between environmental factors and the prokaryotic community distribution

**Figure 4:**
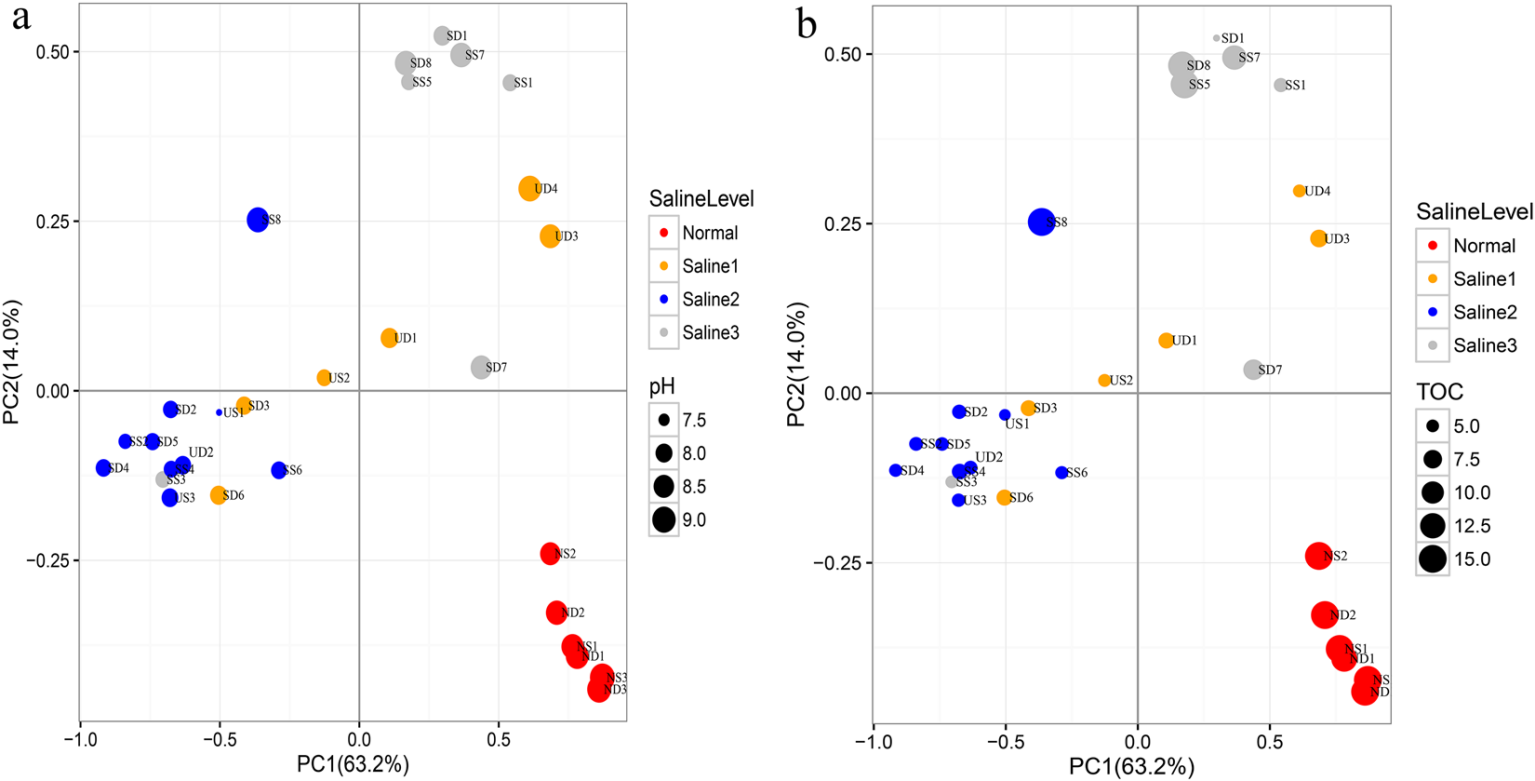
Principal coordinate analyses (PCoA) based on the weighted Unifrace distance of all soil samples. The weighted Unifrace distance was calculated with the normalized OTU table (47504 sequences per sample). Soil samples were distinguished according to their salinity and pH (**a**), salinity and TOC (**b**). Four groups represented different saline level: Normal (six normal soils), Saline1 (EC 17–55 dS/m), Saline2 (EC 55–95 dS/m), Saline3 (> 95 dS/m).

**Figure 5:**
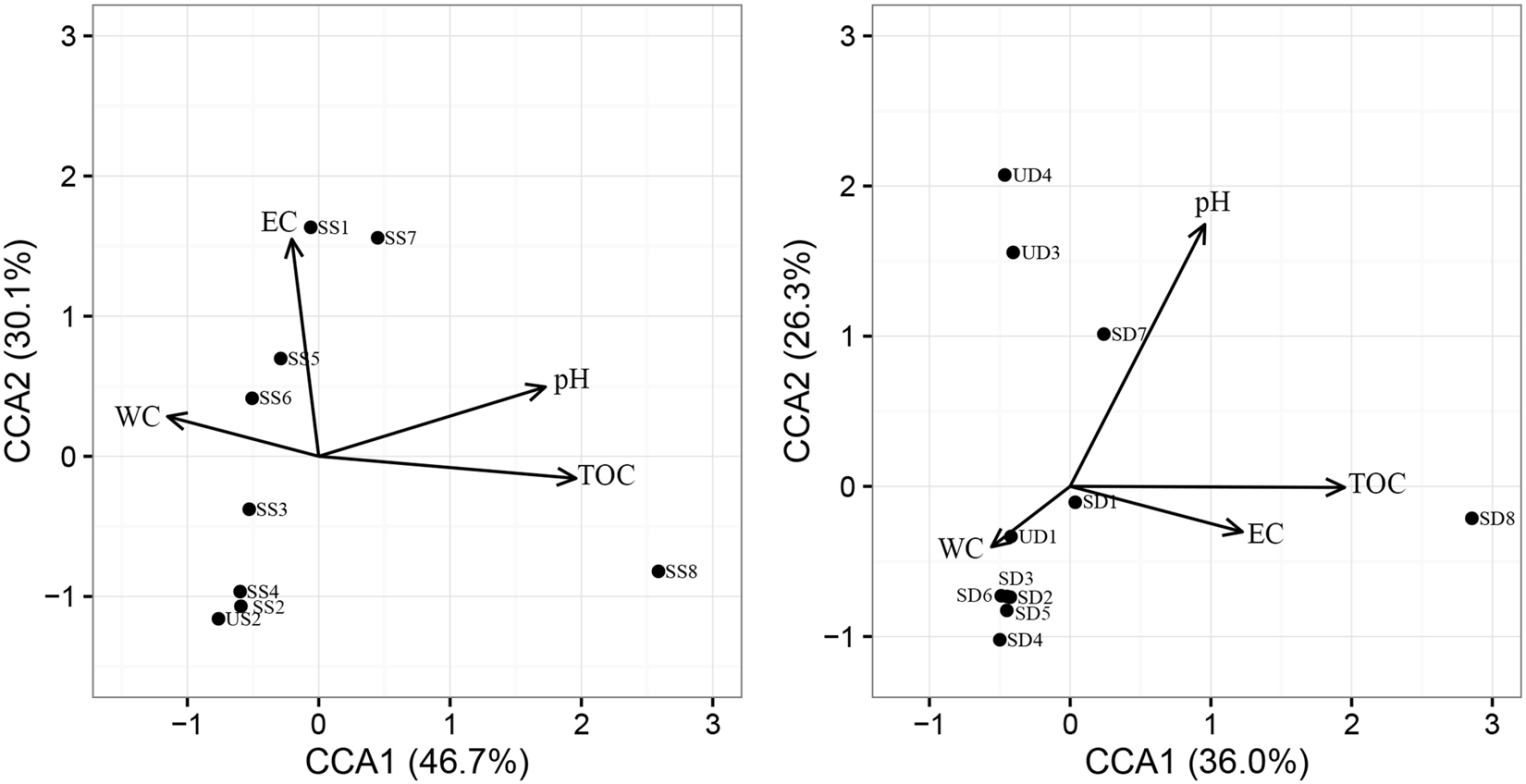
Canonical correlation analysis (CCA) based on the normalized OTU table (47504 sequences per sample) of surface saline soils (**a**) and subsurface saline soils (**b**).

Two main axes explain 77.2% of the soil community variations, and soil samples can be clustered together by both the sizes and colors of points, indicating salinity, pH, and TOC can all affect the prokaryotic community distribution (Fig. 4). CCA biplots showed that pH, TOC, and EC were main factors that affected the prokaryotic community in saline soils: TOC was the strongest factor in surface saline soils (Fig. 5a), so was pH in subsurface saline soils (Fig. 5b).

To further verify the main drivers of the prokaryotic community distribution, we conducted Mantel test between the prokaryotic community and each environment factor of saline soils in Qarhan Salt Lake (Table 2). Only EC, pH, and TOC had significant (*P* < 0.05) correlations with the prokaryotic community distribution in all saline soils. TOC and pH affected the prokaryotic communities in both surface saline soils and subsurface saline soils significantly, but EC only had significant effects on prokaryotic communities in subsurface saline soils (Table 2). Moreover, the *r* value of TOC (*r* = 0.54) were higher than that of pH (*r* = 0.49), suggesting TOC was the main driver of the prokaryotic community distribution in surface saline soils, so was pH in subsurface saline soils. In summary, both CCA and Mantel test proved that TOC was the main driver of the prokaryotic community distribution in surface saline soils, and pH was the main driver of that in subsurface saline soils of Qarhan Salt Lake, China.

**Table 2:** Mantel test results between the prokaryotic community and environmental factors in saline soils of Qarhan Salt Lake.

Factors	Values	All	Surface	Subsurface
EC	*r*	**0**.**23**	0.13	**0**.**38**
	*P*	0.02	0.20	0.04
pH	*r*	**0**.**52**	**0**.**49**	**0**.**55**
	*P*	<0.01	0.01	<0.01
TOC	*r*	**0**.**51**	**0**.**54**	**0**.**51**
	*P*	<0.01	0.01	0.01
WC	*r*	0.17	0.24	0.08
	*P*	0.16	0.11	0.38

Significant (*P* < 0.05) correlation was labeled as boldface.

### Salinity effects on the prokaryotic community

Most samples can be clustered together according to their saline level (Fig. 4 and Supplementary Fig. S4). ANOSIM results further verified the significant prokaryotic community difference across different saline levels (*R* = 0.69, *P* < 0.01). *Halobacteriaceae* (belonging to *Euryarchaeota* of *Archaea*) and *Moraxellaceae* (belonging to *Gammaproteobacteria* of *Bacteria*) have deeper red grids than other families, suggesting they are two most abundant families in saline soils (Supplementary Fig. S4). Half of top 40 most abundant families belongs to *Proteobacteria*, indicating the widespread distribution of *Proteobacteria* in soils. Moreover, normal soils apparently had more complex communities and higher evenness than saline soils (Supplementary Fig. S4).

Though TOC and pH were the main drivers, salinity can still strongly affect the prokaryotic community in saline soils. EC have significant correlations with the prokaryotic community distribution in subsurface saline soils but not with that in surface saline soils (Table 2), suggesting that salinity has more effects on subsurface saline soils. The prokaryotic community of different saline level had different compositions (Supplementary Fig. S3): medium saline soils (group S2 and D2) had higher percentages of *Euryarchaeota* and lower percentages of *Proteobacteria*, *Bacteroidetes* than low saline soils (group S1 and D1) or high saline soils (group S3 and D3) in both surface and subsurface soils. Within *Proteobacteria*, lower percentages of *Gammaproteobacteria* were in medium saline soils than in low saline soils or high saline soils for subsurface soils, and the same trend was observed between medium saline soils and high saline soils but not low saline soils for surface soils (Supplementary Fig. S3). That was probably because group S1 of surface soils contained only one soil sample (sample US2) with relatively high salinity (52.1 dS/m), suggesting that it was probably unrepresentative. We also observed significant correlations between salinity and percentages of several taxa across all saline soil samples (Supplementary Fig. S5). Salinity significantly (*P* < 0.05) affects the percentages of *Euryarchaeota* and *Proteobacteria*, though *R* ^2^ values were relatively low: percentage of *Euryarchaeota* peaks at about 85 dS/m; *Proteobacteria* reaches the lowest at also about 85 dS/m (Supplementary Fig. S5a). *Gammaproteobacteria* showed the same trend with *Proteobacteria* (Supplementary Fig. S5b).

## Discussion

The extremely arid desert climate with high evaporation-precipitation ratio and strong sun exposure is observed in the area of Qarhan Salt Lake. These characters made the unique prokaryotic community in soils of Qarhan Salt Lake. Several reports showed that *Bacteria* were numerically dominant relative to *Archaea* in saline soils of the areas they explored^10,28,29^, and there were also reports showing that *Archaea* were dominant^30,31^. In the present study, we found that *Archaea* were dominant in saline soil samples with EC about 58 dS/m to 93 dS/m; *Bacteria* were dominant in soil samples with lower or higher salinity except three outliers (SD6, SD3, SS3) in Qarhan Salt Lake (Fig. 2). Almost all archaeal tags were assigned to *Halobacteria* of *Euryarchaeota*, which agreed with several previous microbial community studies of hypersaline waters^22,32^, saline soils or sediments^10,26,33,34^. Also, saline soil samples in which *Archaea* were dominant has low alkalinity (pH: 7.36–8.02) and low TOC contents (4.19–6.22%) except SS8 (Fig. 2 and Table 1). That agreed with the fact that most halophilic archaeal representatives (mainly *Halobacteria*) isolated from hypersaline lakes are aerobic, neutrophilic, and some are alkaliphilic^35–37^. And, specific membrane structures and catabolic pathways allows *Archaea* to out-compete *Bacteria* when faced with energy limitation, such as low nutrients in extreme conditions^38^.

For *Bacteria* in saline soils of the present study, *Proteobacteria* were the most abundant phylum, and within *Proteobacteria*, *Gammaproteobacteria* were the most abundant class, followed by *Alphaproteobacteria*, *Deltaproteobacteria*, and *Betaproteobacteria*, which was consistent with a previous meta-analysis of sequences in saline soils^39^. Except *Proteobacteria*, *Bacteroidetes* and *Gemmatimonadetes* were the other two most abundant phyla (Supplementary Fig. S1), agreed with previous reports^10,28^.

For *Archaea* in nonsaline soils from the eastern Tibetan Plateau, the *Halobacteria* were dominant only in dry soils; the most abundant phylum was *Thaumarchaeota* ^40^. Moreover, in nonsaline normal soils from Tianjin, China in the present study, *Archaea* only accounts for a small part, agreed with a previous report^41^. The most abundant archaeal phylum was *Halobacteria*, followed by *Thaumarchaeota*. *Proteobacteria*, *Acidobacteria*, and *Actinobacteria* were the three most abundant phyla of *Bacteria* (Supplementary Fig. S1), consistent with the bacterial community composition in Tibetan permafrost soils^42^. For *Proteobacteria* in normal soils, *Alphaproteobacteria* were the most abundant phylum, followed by *Gammaproteobacteria*, *Deltaproteobacteria*, and *Betaproteobacteria*.

Though with relatively high percentage of shared species (about 50%) (Fig. 1), significant community difference was observed, and *Euryarchaeota* contributed the most to the community variations in both surface and subsurface soils between saline soils and nonsaline soils. Because almost all archaeal tags were assigned to *Halobacteria* of *Euryarchaeota*, *Archaea* and *Halobacteria* had similar contributions to *Euryarchaeota*. Nonsaline normal soils had more complex community and higher evenness than saline soils (Fig. 2 and Supplementary Fig. S4). Nonsaline normal soils were also more diverse than saline soils (Supplementary Table S1 and Fig. 3). Keshri J. *et al*. has also found that the community in nonsaline soils was significantly different from that in saline soils and more diverse than saline soils^18^. Those also agreed with and supported the general belief that relatively low microbial diversity existed in “extreme” environments^43^. Within *Proteobacteria*, most saline samples (16/23) were dominant by *Gammaproteobacteria*, and Arit S. de León-Lorenzana *et al*.^44^ has observed a sharp relative abundance decrease of *Gammaproteobacteria* by flooding a saline soil, suggesting *Gammaproteobacteria* was more adaptive to saline environments than other proteobacterial classes. Moreover, it confirmed the widespread distribution of *Halobacteria* of *Archaea*, *Proteobacteria* of *Bacteria* in soils.

We observed trends of higher salinity, lower water contents, and lower pH in surface soils than in subsurface soils according to physical and chemical properties of eight paired surface-subsurface saline soils (Table 1), which was probably caused by strong sun exposure and high evaporation in Qarhan Salt Lake. In addition, we needed to point out that trends of salinity and pH between surface soils and subsurface soils were unsignificant (t-test, *P* > 0.05), and the water contents in subsurface soil were significantly (*P* = 0.048) higher than those in surface soils. Though with variations of environmental factors, microorganisms in surface soils were richer than those in their corresponding subsurface soils (Fig. 3 and Table 1). According to Hollister E. B. *et al*., the water effect on microbial community was primarily due to its influence on soil oxygen concentrations^10^. And another reports suggested the decrease of biomass along the increasing depth was initially caused by redox state (depth of a few centimeters), and further caused by other variables, such as nutrients and stress (the anoxic zone)^45^. Therefore, we hypothesized that low oxygen concentrations, which might be affected by high water contents, caused low microbial richness in subsurface soils (15–30 cm). That also agreed with previous reports^12,13,15^ microorganisms in surface soils were richer than those in their corresponding subsurface soils.

Different prokaryotic diversities were observed, but we observed no significant community difference between surface soils and subsurface soils in both saline soils and normal soils according to the results of ANOSIM and Student’s t-test, contrary to a previous report^11^. Therefore, we hypothesized that the depth of 15–30 cm probably was not deep enough to generate significant community difference from the surface soil (0–10 cm).

In the present study, though saline soils had much fewer diversities than normal soils (Supplementary Table S1, Fig. 3), however we didn’t observe decreasing microbial richness (observed OTUs) with increasing salinity (*r* = 0.015, *P* = 0.947) in saline soils, just as previous studies have observed^22,25,46^. Moreover, salinity had more effects on subsurface saline soils than surface saline soils according to the result of Mantel test (Table 2). That was probably because compared with subsurface soils, surface soils were more easily subject to other environmental factors^47^, such as strong sun exposure, precipitation or oxygen. The prokaryotic community composition in soils with different saline level differed from each other (Supplementary Fig. S3): higher percentage of *Euryarchaeota* and lower percentage of *Proteobacteria*, *Gammaproteobacteria* existed in medium saline soils (group S2 and D2) than in low saline soils (group S1 and D1) or high saline soils (group S3 and D3) for both surface and subsurface soils. Nonlinear fitting curves also verified the same trend: *Proteobacteria* reaches lowest at about EC 85 dS/m contrary to the percentage of *Euryarchaeota* (Supplementary Fig. S5a); *Gammaproteobacteria* showed the same trend with *Proteobacteria* (Supplementary Fig. S5b), which was possibly caused by the large proportion of *Gammaproteobacteria* in *Proteobacteria*. That agreed with a previous study on soils from the former lake Texcoco, which showed more *Gammaproteobacteria* clones were in low (0.65 dS/m) and high (158 dS/m) saline soils than in medium (56 dS/m) saline soils^48^.

Soil samples were well clustered by pH, TOC, and salinity (Fig. 4); pH and TOC all had significant correlations with the prokaryotic community distribution in both surface saline soils and subsurface saline soils (Table 2). Therefore, we can infer that the prokaryotic community in both surface saline soils and subsurface saline soils were affected by similar environmental factors, agreed with a previous research on the bacterial community distribution in nonsaline soils of the western Tibetan Plateau^11^.

Most previous studies focused on the microbial community in saline waters and sediments, and salinity has been characterized as one of the primary factors of microbial community distribution in aquatic environments^7,8,22^. Many previous studies have observed that salinity had the strongest effect on the prokaryotic community distribution in waters^22,23,25^, sediments^26,27,49^. In the two previous reports on saline soils^9,10^, the former showed salinity had the strongest effect on the bacterial community structure, but the latter showed the microbial community distribution was correlated with other factors better, including organic carbon contents, water contents, pH, and phosphorus contents. Also, another report of archaeal communities in saline soils showed that archaeal community structures considering phylogenetic information were correlated well with pH^34^. In the present study, by summarizing the results of PCoA, CCA, and Mantel test, we found that TOC was the main driver of prokaryotic community distribution in surface saline soils; pH was main driver in subsurface saline soils of Qarhan Salt Lake. Therefore, we confirmed that salinity played a more important role on the microbial community distribution in saline waters or saline sediments than in saline soils; the microbial community in saline soils was more sensitive to other environmental factors, such as TOC, pH, or water contents.

## Conclusion

We collected 23 saline soils from playa of Qarhan Salt Lake to clarify the prokaryotic community distribution along several environmental gradients in both surface (0–5 cm) and subsurface (15–30 cm) saline soils. We showed high-resolution differences between saline soils and nonsaline soils in both prokaryotic diversities and communities. And we found that TOC was the main driver of the prokaryotic community distribution in surface saline soils, so was pH in subsurface saline soils; salinity had more effects on subsurface saline soils than surface saline soils. Our finding can provide references for sampling strategy and prokaryotic community compositions in saline soils of arid areas. Also, the distribution patterns of the prokaryotic community and taxa, especially some halophilic taxa (mainly *Halobacteria* of *Archaea*), along the environmental gradients in saline soils provided references for further new gene exploitations, such as some halophilic or halotolerant enzyme genes. At last, further in-depth investigations on saline soil microbial communities in large scale or with greater depth were expected.

## Methods

### Site climate description and the sample collection

Qarhan Salt Lake has a mean annual temperature of 5.33 °C, a mean annual precipitation of 24 mm, and a mean annual evaporation of 3,564 mm, which indicates an extremely arid desert climate. The climate data were achieved from Qarhan meteorologic station.

In summer 2016, 15 playa sites of Qarhan Salt Lake were selected with a minimum distance of 100 m as sampling sites. The sampling strategy was similar to a previous reports^28^: a) maximum reachable sites in the interior playa even without roads; b) different locations with various salinity levels. Most locations of the playa were bare zones dotted with scarce vegetations, including *Lycium ruthenicum* and *Phragmites australis*. For each site, surface soils (0–10 cm) and subsurface soils (15–30 cm) were collected using a metal auger with a 4-cm diameter. For each soil sample, four samples collected in the vertices of 1-meter side square were mixed into a representative sample. If present, stones, solid salt crust, and roots were removed before sampling. Three paired nonsaline normal soils were collected with the same sampling strategy in Water Park, Tianjin, China. The vegetation of the normal soil locations included *Cynodon dactylon*, *Ophiopogon japonicas*, *Setaria leucopila*, etc, and we selected the locations with few grass to avoid the influence of grassroots. Locations and altitudes were recorded using a GPS. Collected soils were placed into 50 ml sterile plastic centrifuge tubes and stored at 4 °C during transportation. For each sample, one portion was air dried, 2 mm sieved, then physically and chemically analyzed; the other portion was immediately stored at −80 °C for further DNA extraction.

### Physical and chemical analysis

The pH was measured in a 1:2.5 (*w/w*) aqueous solution with a pH meter (PHS-3C; INESA, Shanghai, China). The electrical conductivity (EC) was measured in a 1:5 aqueous solution with a conductivity meter (FE-30; Mettler Toledo, Switzerland). The water content was detected by oven drying fresh soil to a constant weight at 105 °C. Potassium dichromate heating oxidation method^50^ was used to determine the total organic carbon content (TOC). The concentrations of Mg, K, Na, Ca were measured with an atomic absorption spectroscopy (TAS-990; PERSEE, Beijing, China).

### DNA extraction and sequencing

PowerSoil DNA Extraction Kit (Mo Bio Laboratories, CA, USA) was used to extract DNA from soils (about 0.25 g) following the manufacturer’s instructions. But we failed to extract enough DNA from seven samples among all 15 paired saline soil samples due to the low biomass in saline soils. And for the seven failures, we have tested three different kinds of soil weight (0.15 g, 0.25 g, and 0.5 g), all resulting in no success. Primers 515 F and 806R^41^, fused with a barcode and Illumina adaptor, were used to amplify V4 regions of prokaryotic 16S rRNA genes. The PCR products were sequenced using Illumina HiSeq. 2500 platform^51^ (250 bp pair-end reads) by Novogene (Beijing, China).

### Analysis of Illumina-sequencing data

Barcodes and primers of generated Illumina-sequencing reads were removed, and the reads were merged using FLASH software^52^, generating raw tags. Raw tags were qualified using QIIME (version 1.9.1)^53^ software package: a) raw tags, which had three or more consecutive low-quality bases (the threshold value was 19), will be truncated at the first low-quality base; b) some tags, whose consecutive high-quality base length is shorter than 75% of tags length, were further filtered. Then, chimeras were identified and filtered through blasting against the ChimeraSlayer reference database^54^ by UCHIME Algorithm^55^. The effective tags were finally obtained. The obtained effective tags were used for further OTU (operational taxonomic unit) picking and diversity analysis with QIIME. OTUs were picked using the preferred open-reference^56^ OTU picking strategy in QIIME, which includes both close-reference and *de novo* OTU picking strategy; USEARCH^57^ was used to cluster sequences with ≥ 97% similarity together forming an OTU. A representative sequence was picked out from each OTU, and the taxonomy was assigned to each of the representative sequences with Ribosomal Database Project (RDP) classifier^58^ against Greengenes^59^ at a confidence threshold of 0.8 in QIIME. Representative sequences were aligned against the Greengenes core set with PyNAST^60^, then a phylogenetic tree was generated with FastTree^61^, which was used to calculated the weighted UniFrac distance^62^.

Before diversity analysis, the sequences of each sample were normalized to 47504 tags based on the number of tags in the sample with the fewest tags. Several alpha diversity indices, which includes observed OTUs, Chao1 richness estimator, and Shannon diversity index, were calculated with QIIME. Principal Coordinate Analysis (PCoA) based on weighted Unifrac distance matrix was conducted with the function “cmdscale” of package Stats in R (version 3.3.1). Heat map was drawn using the package Pheatmap in R. Canonical correlation analysis (CCA), analysis of similarity (ANOSIM), similarity percentage (SIMPER) analysis^63^, and Mantel test were conducted with the package Vegan^64^ in R. We chose CCA according to detrended correspondence analysis (DCA) and removed environmental factors with variance inflation factors of more than ten from CCA to reduce effects of the collinearity. Mantel test was performed to analyze the correlation between the prokaryotic community composition (Bray-Curtis distance based on OTU-level communities) and each environmental factor (Euclidean distance). ANOSIM was used to evaluate significant differences of the prokaryotic community composition among different groups.

### Data availability

The original sequences of this research were deposited at the NCBI Sequence Read Archive under the accession number SRP108198. The accession number of each sample was also listed (Supplementary Table S1).

## Electronic supplementary material

Supplementary Infromation
