# Supplementary material for: Prokaryotic Community Distribution along an Ecological Gradient of Salinity in Surface and Subsurface Saline Soils
Source: Sci Rep. 2017 Oct 17;7:13332. doi: 10.1038/s41598-017-13608-5 (PMC5645410; doi:10.1038/s41598-017-13608-5)
Supplement: Supplementary file 1 — Supplementary Infromation [file 41598_2017_13608_MOESM1_ESM.pdf]

Prokaryotic Community Distribution along an Ecological Gradient of Salinity in Surface and Subsurface Saline Soils

Kehui Xie[1], Yong Deng[1], Shaocun Zhang[1], Wenhao Zhang[1], Jianrong Liu[2], Yulong Xie[2], Xuze Zhang[2]* & He Huang[1]*

[1]School of Chemical Engineering and Technology, Tianjin University, Tianjin, 300350, People's Republic of China.
[2]School of Chemistry and Chemical Engineering, Qinghai University for Nationalities, Xining, 810007, People's Republic of China.
*To whom correspondence should be addressed: H.H. (huang@tju.edu.cn) or X.Z. (zxz1904@126.com)

Supplementary Tables

Table S1. Statistics and alpha diversity indices of sequencing data

Type	Sample ID	Accession no.	Raw tags	Effective tags	Ave Length (bp)	OTUs	Chao1	Shannon	Q20	Q30	GC%
Saline soils	SS1	SRS2237005	64,255	62,916	253	2174	4318	3.4	99.43	98.86	54.18
	SD1	SRS2237021	52,464	51,487	253	2549	5060	5.02	99.38	98.77	55.24
	SS2	SRS2237023	68,089	67,162	254	1739	3455	5.3	99.33	98.63	60.49
	SD2	SRS2237025	49,755	48,838	254	2438	4756	6.91	99.41	98.8	60.38
	SS3	SRS2237026	58,622	57,252	254	2957	5494	7.11	99.38	98.73	59.85
	SD3	SRS2237133	66,007	64,779	254	2457	5109	7.12	99.4	98.75	60.34
	SS4	SRS2237134	63,906	62,756	254	2516	4754	6.35	99.34	98.65	60.05
	SD4	SRS2237135	61,808	60,839	254	2053	4141	6.09	99.4	98.78	60.86
	SS5	SRS2237136	50,256	48,467	253	2749	4838	5.73	99.39	98.78	55.96
	SD5	SRS2237160	55,485	54,523	254	2328	4673	7.08	99.34	98.63	60.89
	SS6	SRS2237161	56,332	54,586	253	4857	8252	8.72	99.36	98.71	58.26
	SD6	SRS2237162	50,801	49,425	254	3034	5354	7.77	99.36	98.7	59.24
	SS7	SRS2237163	66,454	65,012	253	2472	4761	5.13	99.26	98.65	55.73
	SD7	SRS2237164	61,149	60,296	253	2215	4303	5.16	99.44	98.84	60.47
	SS8	SRS2237165	63,714	62,165	254	874	1130	5.51	99.13	98.23	58.6
	SD8	SRS2237166	67,855	66,440	255	584	749	2.8	99.29	98.53	56.14
	US1	SRS2237167	53,615	52,366	253	2294	4057	5.84	99.4	98.77	58.09
	US2	SRS2237168	49,772	48,608	253	2677	4868	7.68	99.35	98.66	59.55
	US3	SRS2237169	68,596	67,321	254	3210	6227	5.35	99.36	98.7	60.01
	UD1	SRS2237170	67,308	65,919	253	3655	6907	6.9	99.36	98.67	60.28
	UD2	SRS2237171	53,992	52,951	254	3402	6901	6.51	99.29	98.57	59.4
	UD3	SRS2237172	48,258	47,504	253	1189	2317	3.19	99.09	98.39	57.11
	UD4	SRS2237173	65,490	64,038	254	2118	3842	5.61	99.33	98.68	54.64
Normal soils	NS1	SRS2237174	66,419	63,795	253	6549	10191	10.71	99.32	98.64	57.03
	ND1	SRS2237175	63,260	60,906	254	6025	9253	10.38	99.29	98.56	57.66
	NS2	SRS2237176	67,240	64,426	255	5779	8789	9.99	99.27	98.54	56.96
	ND2	SRS2237177	52,894	51,102	254	5513	8825	10.08	99.39	98.78	57.39
	NS3	SRS2237353	57,842	55,646	254	5117	7732	9.98	99.31	98.63	56.78
	ND3	SRS2237354	64,761	62,366	253	4894	7517	9.97	99.33	98.66	57.33

Table S2. Spearman rank correlations between EC and other environmental factors. Significant ($P < 0.05$) correlation was labeled as boldface.

Factors	*r* value	*P* value
K	**-0.48**	0.01
Na	**0.86**	<0.01
Ca	**0.48**	0.01
Mg	**0.77**	<0.01
pH	**-0.47**	0.01
TOC	**-0.50**	0.01
WC	**-0.53**	0.01

Table S3. Relative abundance of ten most predominant phyla/classes in all soils. Samples were sorted from top to bottom with increasing salinity.

	Sample	*Euryar-chaeota*	*Gamma-proteobacteria*	*Alpha-proteobacteria*	*Delta-proteobacteria*	*Beta-proteobacteria*	*Gemma-timonadetes*	*Bacte-roidetes*	*Acido-bacteria*	*Actino-bacteria*	*Plancto-mycetes*
Normal soils	NS3	0.0007	0.1262	0.1322	0.0661	0.0574	0.0755	0.0402	0.2312	0.1251	0.0360
	NS2	0.1157	0.1606	0.1574	0.0491	0.0317	0.0685	0.0246	0.1845	0.1111	0.0331
	ND2	0.0901	0.1335	0.1214	0.0616	0.0451	0.0948	0.0278	0.1852	0.1066	0.0415
	ND3	0.0006	0.1075	0.1329	0.0718	0.0601	0.0856	0.0248	0.1804	0.1484	0.0382
	ND1	0.0364	0.1195	0.1273	0.0770	0.0501	0.1182	0.0666	0.1697	0.1029	0.0300
	NS1	0.0637	0.1192	0.1404	0.0724	0.0528	0.0579	0.0365	0.1861	0.1109	0.0481
Saline soils	UD3	0.1231	0.6590	0.0055	0.0113	0.0003	0.0022	0.1709	0.0002	0.0242	0.0002
	SD6	0.6557	0.0477	0.1236	0.0171	0.0037	0.0151	0.0238	0.0028	0.0853	0.0029
	UD4	0.1605	0.5728	0.1251	0.0130	0.0215	0.0106	0.0413	0.0005	0.0288	0.0001
	SD3	0.5970	0.0429	0.0938	0.0152	0.0042	0.0768	0.1179	0.0008	0.0067	0.0013
	UD1	0.3498	0.0513	0.0444	0.0338	0.0091	0.2053	0.2325	0.0154	0.0188	0.0041
	US2	0.4486	0.0803	0.1768	0.0134	0.0098	0.1169	0.0890	0.0007	0.0063	0.0003
	SD4	0.8665	0.0128	0.0222	0.0061	0.0004	0.0524	0.0221	0.0007	0.0043	0.0007
	US1	0.6383	0.0298	0.2749	0.0048	0.0217	0.0037	0.0022	0.0070	0.0068	0.0009
	UD2	0.7321	0.0865	0.0599	0.0194	0.0060	0.0204	0.0152	0.0163	0.0198	0.0021
	SD2	0.7742	0.0893	0.0406	0.0065	0.0018	0.0104	0.0221	0.0006	0.0133	0.0001
	SS2	0.8384	0.0605	0.0168	0.0029	0.0012	0.0383	0.0142	0.0002	0.0047	0.0000
	SS8	0.5921	0.3143	0.0001	0.0000	0.0000	0.0000	0.0845	0.0000	0.0013	0.0000
	US3	0.7553	0.0631	0.0353	0.0143	0.0080	0.0449	0.0190	0.0153	0.0215	0.0031
	SS4	0.7249	0.0587	0.0771	0.0109	0.0028	0.0591	0.0425	0.0008	0.0057	0.0007
	SD5	0.7938	0.0470	0.0104	0.0118	0.0010	0.0629	0.0533	0.0004	0.0037	0.0003
	SS6	0.5557	0.0940	0.0783	0.0401	0.0153	0.0257	0.0443	0.0238	0.0353	0.0051
	SD7	0.2146	0.0607	0.0155	0.0072	0.0035	0.3606	0.1975	0.0118	0.0992	0.0030
	SS7	0.2893	0.5277	0.0192	0.0066	0.0087	0.0085	0.0844	0.0134	0.0334	0.0013
	SS1	0.2089	0.6658	0.0400	0.0074	0.0106	0.0135	0.0049	0.0170	0.0203	0.0014
	SD8	0.3696	0.6082	0.0006	0.0014	0.0004	0.0000	0.0049	0.0000	0.0009	0.0000
	SS3	0.7640	0.0329	0.0592	0.0394	0.0053	0.0150	0.0286	0.0018	0.0196	0.0006
	SS5	0.3397	0.5255	0.0308	0.0104	0.0025	0.0272	0.0407	0.0008	0.0081	0.0002
	SD1	0.3362	0.5419	0.0221	0.0094	0.0096	0.0098	0.0094	0.0160	0.0175	0.0015

Table S4. SIMPER community analysis between saline soils and normal soils.

	surface normal soils VS surface saline soils					subsurface normal soils VS subsurface saline soils				
Phyla	AveN	AveS	AveCon	SD	ord	AveN	AveS	AveCon	SD	ord
Euryarchaeota	0.060	0.539	0.24	0.115	1	0.042	0.576	0.267	0.117	1
Gammaproteobacteria	0.135	0.285	0.112	0.09	2	0.120	0.181	0.083	0.086	9
Alphaproteobacteria	0.143	0.040	0.052	0.015	9	0.127	0.041	0.043	0.021	2
Deltaproteobacteria	0.063	0.015	0.024	0.009	3	0.070	0.009	0.030	0.004	7
Betaproteobacteria	0.047	0.006	0.021	0.006	10	0.052	0.003	0.024	0.003	10
Proteobacteria_others	0.004	0.005	0.003	0.005	4	0.003	0.001	0.001	0.000	3
Gemmatimonadetes	0.067	0.023	0.022	0.01	7	0.100	0.073	0.046	0.036	4
Bacteroidetes	0.034	0.043	0.011	0.009	5	0.040	0.056	0.023	0.025	5
Acidobacteria	0.201	0.007	0.097	0.012	11	0.178	0.004	0.087	0.004	8
Actinobacteria	0.116	0.016	0.05	0.007	8	0.119	0.029	0.045	0.022	11
Planctomycetes	0.039	0.001	0.019	0.003	13	0.037	0.001	0.018	0.003	13
Firmicutes	0.009	0.008	0.002	0.002	15	0.007	0.009	0.003	0.003	14
Chloroflexi	0.023	0.004	0.009	0.004	16	0.029	0.002	0.013	0.002	16
Nitrospirae	0.013	0.001	0.006	0.002	14	0.022	0.000	0.011	0.003	15
Verrucomicrobia	0.017	0.001	0.008	0.001	6	0.013	0.000	0.006	0.001	24
Thaumarchaeota	0.015	0.000	0.007	0.005	24	0.019	0.000	0.010	0.005	12
OP1	0.000	0.001	0.001	0.002	12	0.000	0.001	0.000	0.001	20
[Thermi]	0.001	0.001	0	0	19	0.000	0.001	0.000	0.000	19
WS3	0.002	0.000	0.001	0.001	21	0.003	0.000	0.001	0.000	21
Cyanobacteria	0.001	0.000	0	0	17	0.005	0.000	0.002	0.003	6
Armatimonadetes	0.002	0.000	0.001	0	18	0.003	0.000	0.001	0.000	22
TM7	0.001	0.000	0	0	22	0.001	0.000	0.001	0.000	17
TM6	0.000	0.000	0	0	20	0.000	0.000	0.000	0.000	18
Others	0.009	0.003	0.003	0.001	23	0.011	0.011	0.004	0.002	23

Abbreviations: AveN: average relative abundance of phyla/classes in normal soils; AveS: average relative abuntance of phyla/classes in saline soils; AveCon: average contributions of phyla/classes; SD: Standard deviation of the average contribution; ord: orders of phylum/class contributions

Supplementary Figures

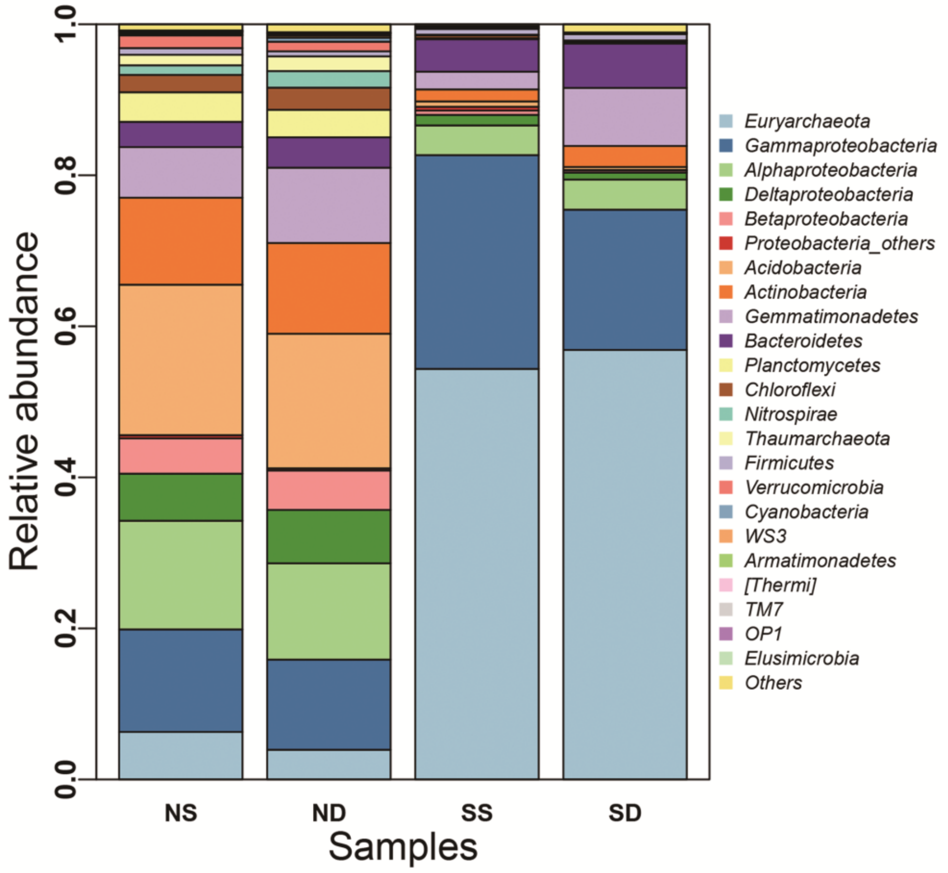

Figure S1. Relative abundance of 19 most predominant phyla (including four classes in *Proteobacteria*) among four groups same as Figure 1 but without seven unpaired samples to remove the influence of environmental factors (same as Figure 3).

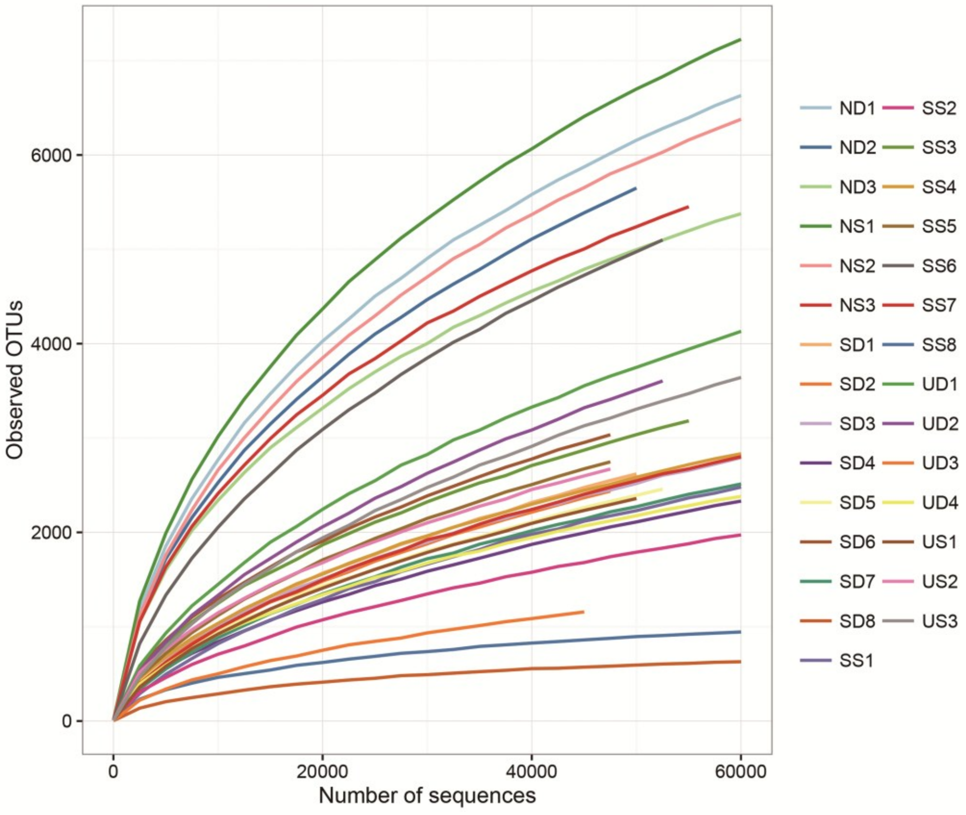

Figure S2. Rarefaction plot for observed OTUs of all samples with QIIME.

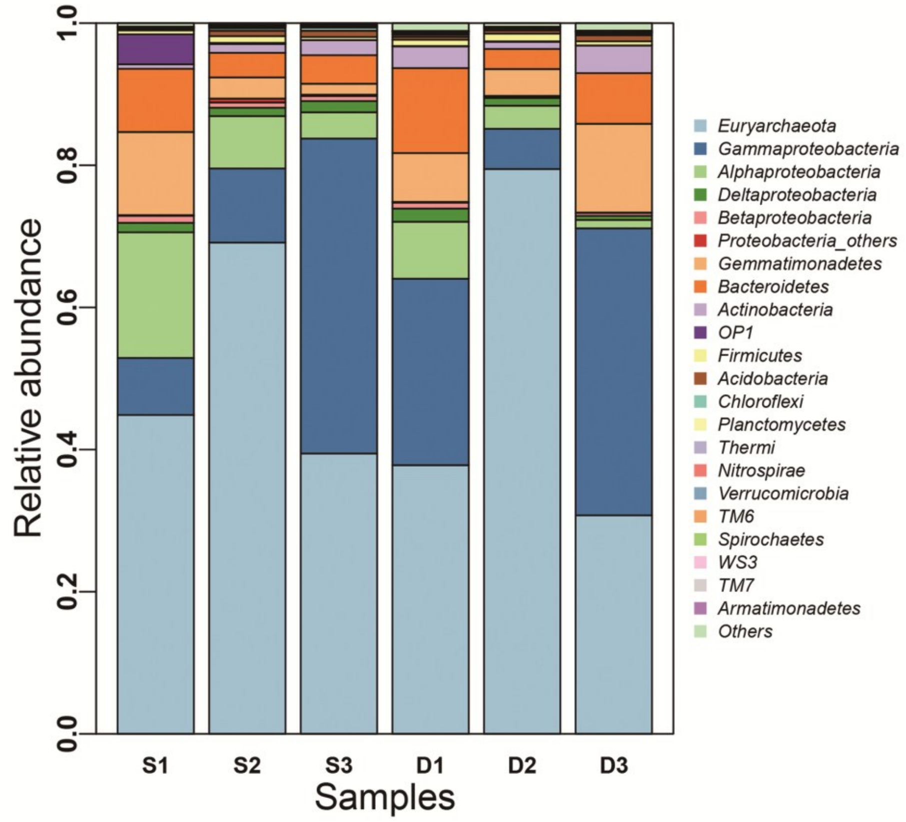

Figure S3. Relative abundance of 18 most predominant phyla (including four classes in *Proteobacteria*). Surface saline soils were divided into three groups (S1, S2, S3) according to their saline level same as Figure 4, so were subsurface saline soils (D1, D2, D3).

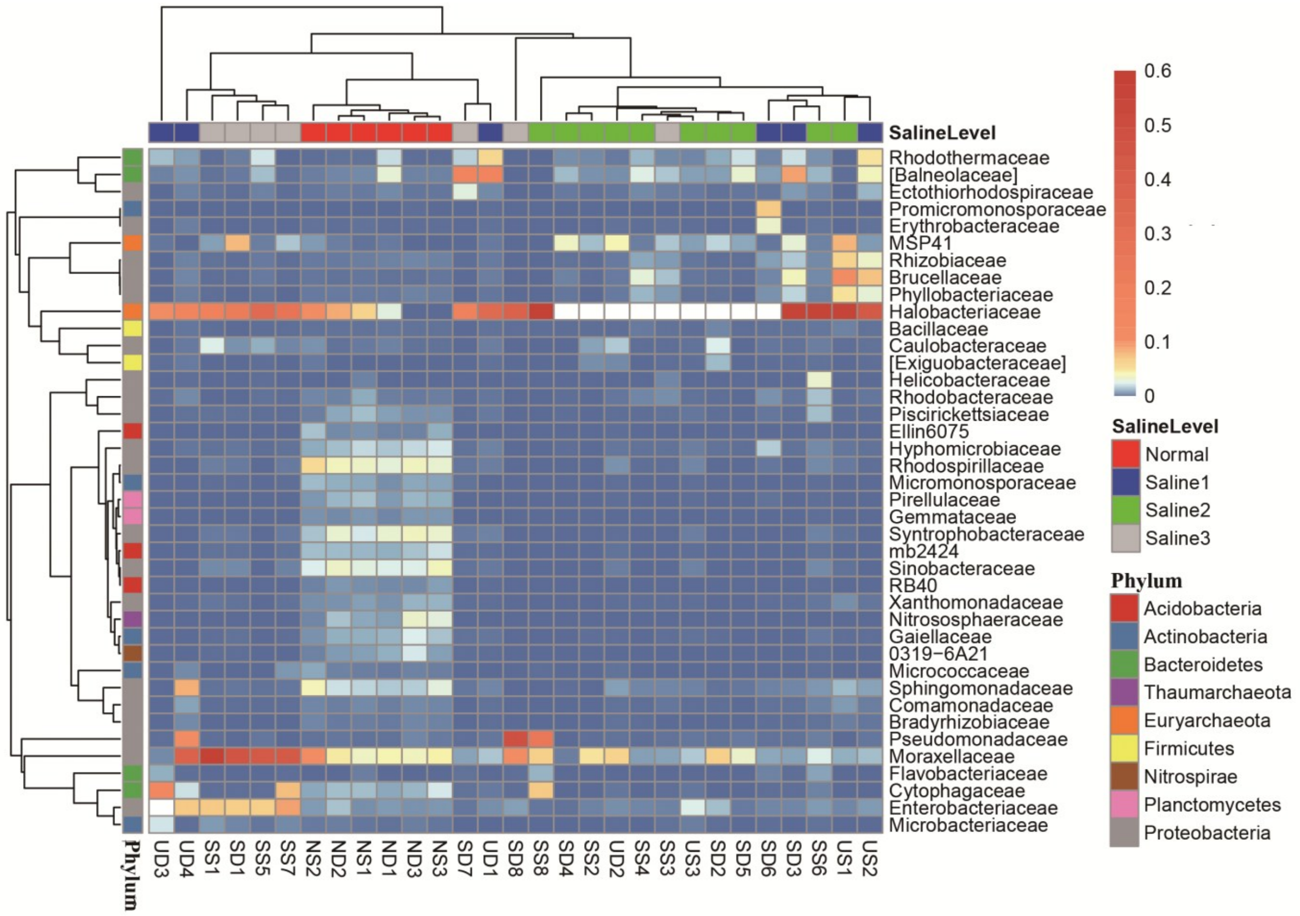

Figure S4. Heat map showing relative abundance of top 40 most abundant families and the hierarchical clustering of all soil samples (Pearson correlation, UPGMA) and taxa (Euclidean, UPGMA). The color code representing relative abundance ranges from blue (low abundance) to red (high abundance).

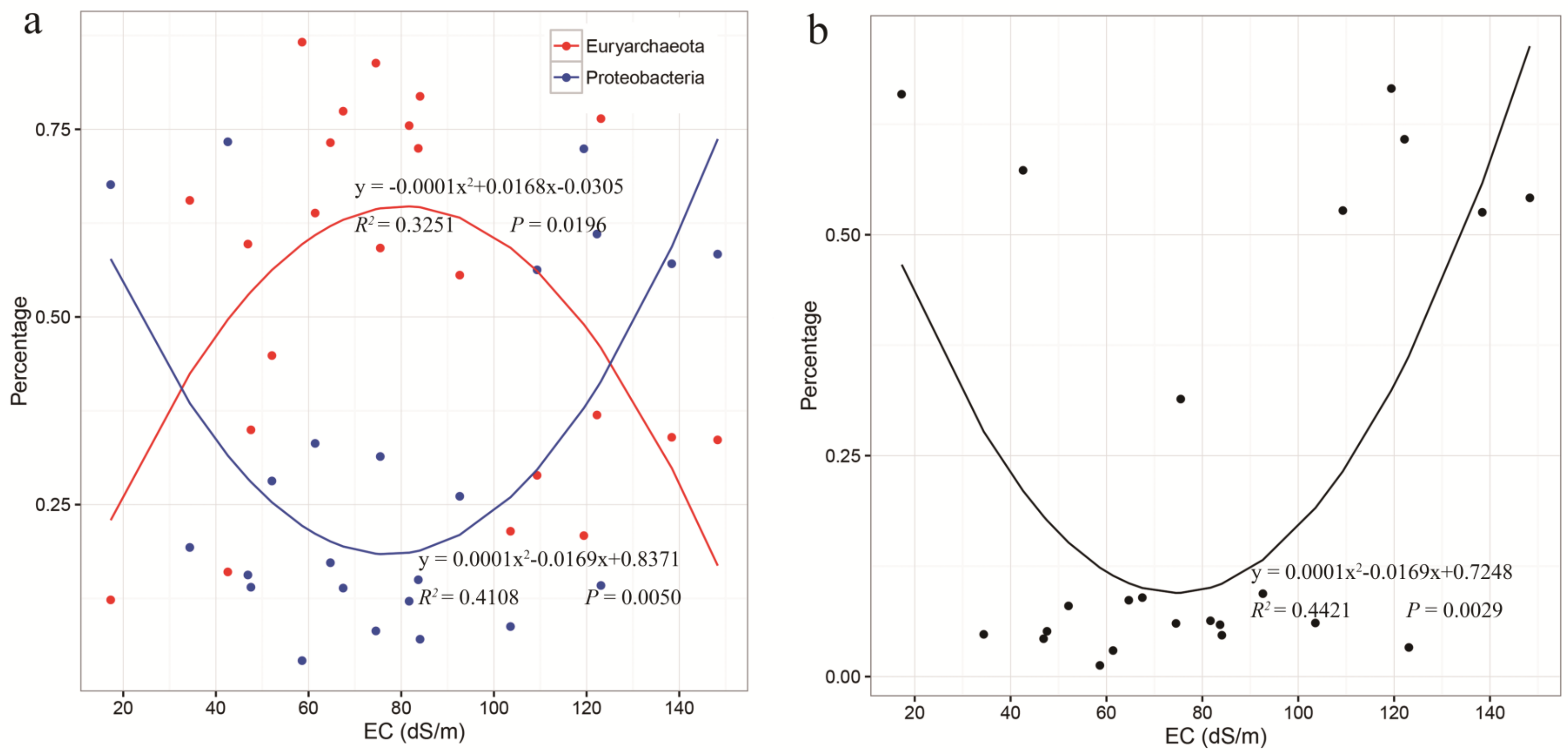

Figure S5. Nonlinear fitting curves between salinity and percentages of *Euryarchaeota* and *Proteobacteria* (a), *Gammaproteobacteria* (b) among saline soils.
